# B-cell epitopes of African horse sickness virus serotype 4 recognised by immune horse sera

**DOI:** 10.4102/ojvr.v84i1.1313

**Published:** 2017-02-24

**Authors:** Evans M. Mathebula, Frederika E. Faber, Wouter van Wyngaardt, Antoinette van Schalkwyk, Alri Pretorius, Jeanni Fehrsen

**Affiliations:** 1 New Generation Vaccines Programme, Agricultural Research Council - Onderstepoort Veterinary Institute, South Africa; 2 Department of Veterinary Tropical Diseases, University of Pretoria, South Africa; 3 Molecular Epidemiology and Diagnostics, Agricultural Research Council - Onderstepoort Veterinary Institute, South Africa

## Abstract

Identifying antigenic proteins and mapping their epitopes is important for the development of diagnostic reagents and recombinant vaccines. B-cell epitopes of African horse sickness virus (AHSV) have previously been mapped on VP2, VP5, VP7 and NS1, using mouse, rabbit and chicken monoclonal antibodies. A comprehensive study of the humoral immune response of five vaccinated horses to AHSV-4 antigenic peptides was undertaken. A fragmented-genome phage display library expressing a repertoire of AHSV-4 peptides spanning the entire genome was constructed. The library was affinity selected for binders on immobilised polyclonal immunoglobulin G (IgG) isolated from horse sera collected pre- and post-immunisation with an attenuated AHSV-4 monovalent vaccine. The DNA inserts of binding phages were sequenced with Illumina high-throughput sequencing. The data were normalised using pre-immune IgG-selected sequences. More sequences mapped to the genes coding for NS3, VP6 and VP5 than to the other genes. However, VP2 and VP5 each had more antigenic regions than each of the other proteins. This study identified a number of epitopes to which the horse’s humoral immune system responds during immunisation with AHSV-4.

## Introduction

African horse sickness virus (AHSV) is a member of the genus *Orbivirus* in the family *Reoviridae* (Calisher & Mertens 1998). It contains 10 linear double-stranded RNA segments which code for seven structural (VP1–7) and at least five non-structural proteins (NS1–4, S10-ORF2; Belhouchet et al. 2011; Roy, Mertens & Casal 1994; Stewart et al. 2015; Zwart et al. 2015). The gene segment coding for VP6 (segment 9) also codes for NS4, while segment 10 encodes NS3/3A and S10-ORF2. The structural proteins are arranged in three concentric layers, with VP2 and VP5 forming the outer capsid and are associated with cell attachment and inducing apoptosis (Manole et al. 2012; Vermaak & Theron 2015). The inner capsid consist of VP7 and VP3. It encloses the minor structural proteins VP1, VP4 and VP6 (replication complex) and the genome itself (Maree & Paweska 2005; Mellor & Hamblin 2004). There is currently no cure for AHS and the disease is controlled by vaccination. Although the vaccine is effective, there is the potential that the attenuated vaccine may revert to virulence. In addition, infected animals cannot be differentiated from vaccinated animals, a factor which is important in non-endemic areas (Kanai et al. 2014). There is thus a need for alternative AHS vaccines. Although inactivated and subunit vaccines have also been developed, they are not yet commercially available (Lelli et al. 2013; Roy et al. 1996; Scanlen et al. 2002).

Vaccination results in a predominantly humoral response with neutralising antibodies conferring protection (Burrage et al. 1993). Recombinant AHSV VP2 and VP5 alone or in combination induce protection in horses that is enhanced by the addition of VP7 (Alberca et al. 2014; Calvo-Pinilla et al. 2014, 2015; Kanai et al. 2014; Martinez-Torrecuadrada et al. 1996). Understanding the immunogenicity of AHSV proteins and mapping their critical epitopes can contribute to developing an alternative recombinant vaccine. Some B-cell antigenic regions on VP2, VP5 and NS1 of AHSV-4 have already been mapped using a variety of methods including phage display and PEPSCAN (Bentley et al. 2000; De la Poza et al. 2015; Martinez-Torrecuadrada et al. 1999, 2001). This study focusses on B-cell epitopes mapped by phage display technology using sera from horses vaccinated with attenuated AHSV-4.

Phage display of peptides involves constructing a library of fragments of a gene or genome of interest fused to a gene encoding a phage surface protein (Smith & Petrenko 1997). The library of phage-displayed fusion peptides is then iteratively amplified and screened for target binders. Traditionally, after a few rounds of selection, about 10–100 clones are picked and insert DNA sequenced to identify the enriched peptides. This approach was used to map antigenic regions on VP7 (Du Plessis et al. 1994), VP5 (Wang et al. 1995) and NS1 (Du Plessis, Romito & Jordaan 1995) of bluetongue virus (BTV) and AHSV VP2 (Bentley et al. 2000). Owing to the small sample, there is a possibility that not all binding clones are identified when picking a subset. Depending on the diversity of the clones after panning, clones that were enriched may not be among those picked for sequencing or tested for binding (Domina et al. 2014; Ngubane et al. 2013). Using high-throughput sequencing, the entire pool of phages released after panning identifies all the selected peptides. During panning, peptides that do not bind the target of interest are often present. These are termed target-unrelated peptides (TUPs) and should be considered when analysing results (Vodnik et al. 2011). Using appropriate controls during panning, these sequences are used to normalise the experimental samples and the TUPs are removed. In conjunction with phage display, the large amount of data obtained from high-throughput sequencing has allowed rapid profiling of antigenic regions on the meningococcal virulence factor (*Neisseria* adhesin A) by antibodies from vaccinated people (Domina et al. 2014). This technique has been termed PROFILER (Phage-based Representation OF Immuno-Ligand Epitope). In other similar studies, using random peptide libraries in combination with high-throughput sequencing, peptides that bind to *Mycobacterium tuberculosis* (Ngubane et al. 2013) and patient-specific epitope motifs in serum from patients with peanut allergies (Christiansen et al. 2015) have been identified.

In the present study, phage display and high-throughput sequencing were used to dissect the immunogenicity of a live attenuated AHSV-4 vaccine in horses. The B-cell epitopes encoded by all 10 AHSV-4 segments as recognised by polyclonal antibodies from immunised horses were analysed. The epitopes identified here could be useful in diagnostic reagent development and, in conjunction with T-cell epitopes identified in a parallel study, have the potential to be included in an envisaged multi-epitope vaccine.

## Materials and methods

### Horse serum samples

Horse sera were obtained from a vaccine trial where five horses (Horse 1–5) were each vaccinated twice subcutaneously with the live-virus–attenuated AHSV-4 vaccine strain (Pretorius, Faber & Van Kleef 2016). The research protocol was approved by both the animal ethic committees of the Agricultural Research Council - Onderstepoort Veterinary Institute (ARC-OVI) and Onderstepoort Biological Products (OBP), Ltd. Approval was also obtained from the South African Department of Agriculture, Forestry and Fisheries. Blood was collected for serum 3 days before vaccination (day 0), 7 days after the booster inoculation on day 21 (day 28) and 31 days later (day 52). Immunoglobulin G (IgG) was purified from the sera using the Protein G HP spin trap kit (GE Healthcare Life Sciences) following manufacturer’s protocol.

### Enzyme-linked immunosorbent assay of horse sera samples

To check whether the vaccine induced a humoral response, the horse antisera were tested for the presence of anti–AHSV-4 antibodies. A 96-well microtiter plate (Nunc Polysorp) was coated with 50 µL of 10 µg/mL sucrose-purified AHSV-4 particles (Huismans et al. 1987) diluted in PBS (pH 7.4). The plate was incubated at 4 °C overnight, emptied and blocked with 2% milk powder in PBS (MPBS). Wells coated with MPBS only served as negative control. The plate was incubated for an hour at room temperature. Three washes with PBS containing 0.05% Tween-20 (PBST) were performed after every incubation. After washing, 50 µL of horse serum diluted 200x in MPBS was added to duplicate wells. This was incubated at 37 °C for an hour. After washing, 50 µL of Protein A/G peroxidase (Thermo Scientific) diluted 1:10 000 in MPBST was added to each well and incubated at 37 °C for 45 min followed by washing. Fifty microliters of substrate consisting of one o-phenylenediamine dihychloride tablet (Sigma) dissolved in 5 mL of 0.1 M citrate buffer (pH 4.5) and 2.5 µL of 30% (v/v) hydrogen peroxide (Saarchem) was added. The reaction was stopped after 10 min at room temperature by adding 50 µL of 2 N H_2_SO_4_. The absorbance values were recorded at 492 nm.

### Fragmented-genome phage display library construction

This library was constructed essentially as previously described (Du Plessis & Jordaan 1996; Fehrsen et al. 2005; Gupta et al. 1999) by using existing cDNA copies of each AHSV-4 genome segment cloned into either pET102D-TOPO or pGEMT (Faber et al. 2016). Large segments encoding VP1, VP2 and VP3 were cloned as two fragments. The segments were amplified from these plasmids using TaKaRa Ex Taq enzyme with PCR primer pairs T7a: TAGTTATTGCTCAGCGGTGG / TrxFus: TTCCTCGACGCTAACCTG (for pET102/D-TOPO) and T7b: TAATACGACTCACTATAGGG / M13rev: CAGGAAACAGCTATGAC (for pGEM-T). Annealing temperatures were 56 °C and 54 °C, respectively. DNase 1 digestion of four pools of similar sized amplicons yielded DNA fragments of 50 bp – 600 bp that were gel purified using a QIAquick Gel Extraction kit (QIAGEN) before cloning in the *Pme1* linearised M13 phagemid vector pCVEP1585042 (Fehrsen et al. 2005). After electroporation of the phagemids into *Escherichia coli* TG1 electroporation-competent cells (Agilent Technologies), the presence of inserts was confirmed by using PCR primers M13Ff: GTAAAACGACGCGCAG and M13Rev: CAGGAAACAGCTATGAC, while high-throughput sequencing was used to confirm full-genome representation in the library.

Phages were rescued for panning from bacterial cells containing the phagemids as described previously (Van Wyngaardt & Du Plessis 1998).

### Affinity screening

The library was screened for binders with purified IgGs from horse sera collected on days 0, 28 and 52. The panning process was carried out as previously described (Fehrsen et al. 2005) with minor modifications. Wells of a microtiter plate (Polysorp, Nunc) were coated, in duplicate, with 100 µL of 20 µg/mL of IgG diluted in PBS, for each time point of the five horses separately. The plate was incubated overnight at 4 °C. The next day, unbound IgGs were discarded and the wells blocked with MPBS at room temperature for an hour. The wells were then washed three times with PBS. During this time, 1.12 × 10^12^ fusion phages from the library were pre-incubated for 20 min in 1 mL of MPBS containing 0.1% Tween-20. After washing with PBS, 100 µL of the pre-incubated phages were added to each well and incubated at 37 °C for 1 hour. Unbound phages where removed by washing the wells 10 times with 0.1% PBST and another 10 times with PBS. The bound phages were eluted with 100 µL of 0.1 M glycine–HCl (pH 2.2) elution buffer at room temperature for 10 minutes. The released phages were transferred to a new tube containing 50 µL of 1 M Tris–HCl (pH 9.0) neutralising buffer. The phages were used to infect exponentially growing TG1 cells with an OD_600_ of 0.5–0.6. This was incubated at 37 °C for 30 min and another 30 min shaking at 100 rpm. Ten-fold dilutions of the infected cells were made and 100 µL of each dilution plated on TYE containing 100 µg/mL ampicillin and 2% glucose plates. The remaining cells were centrifuged at 2000 g for 15 min, the pellet resuspended in PBS and 200 µL plated on similar 150-mm plates. The plates were incubated at 30 °C overnight and the number of output colonies recorded. Colonies were scraped from the plates and phages rescued as above. After each round, half the rescued phages were used as input for the subsequent round and the remainder stored at -70 °C. These polyclonal pools of phages were tested in enzyme-linked immunosorbent assay (ELISA) (Van Wyngaardt et al. 2004) for binding to the IgG coated as for screening. Four rounds of panning were performed. Phagemid DNA was isolated from the bacteria after each round and inserts were amplified by PCR for sequencing.

### High-throughput sequencing

DNA inserts were amplified from phagemids using TaKaRa Ex Taq enzyme at a Tm of 56 °C and primers Ff: CGT CGGCAGCGTCAGATGTGTATAAGAGACAGGAG GCTAGCAACGCGTCG and Rev: GTCTCGTGGGCTCG GAGATGTGTATAAGAGACAGCCAGGCGCGCCG. They bind very close to the insert and contain the Illumina MiSEQ sequencing platform adapters (underlined). High-throughput sequencing on gel-purified amplicons was done by Inqaba Biotech (South Africa) using the Illumina MiSEQ v3 (150 cycles) platform.

### Bioinformatic analysis of selected sequences

The high-throughput paired-end sequencing data were analysed using the CLC genomics workbench v7.5 (http://www.clcbio.com/products/clc-genomicsworkbench/) and Microsoft^®^ Excel 2013. Some fragments in the pool were represented by a single read where the second read did not meet the quality standard. After primer sequences were trimmed, low quality and sequences less than 50 bp were discarded. The sequences were mapped to a list containing the open reading frames (ORFs) of all 10 AHSV-4 genome segments. The mapping profile of each segment was analysed individually for each horse.

### Sequence distribution

The sequences selected by immune IgG were normalised by subtracting the pre-immune IgG-selected sequences. To get an overall view of the reads mapping to each genome segment, data were converted to percentages: (number of reads mapping to a segment / total number of reads mapping the entire genome) × 100. The sequences were then further analysed by showing the position of each mapped sequence on the genome segment. For this purpose, the total number of mapped sequences for each segment was exported from CLC to Microsoft^®^ Excel without gaps. This was converted to proportions: total number of base matches per position / total number of base matches on the segment and illustrated by a graph. The data for all the horses were plotted on the same graph. The peaks were numbered with the most important criteria that all horses recognise the region.

Nucleic acid sequences were translated to amino acid sequences to confirm the in-frame display of peptides with the vector-encoded pVIII, which is necessary to produce a functional pVIII and natural AHSV peptides, and also to establish if the amino acid sequence mapping results in any notable change when compared to that using nucleotide sequences. After converting the unpaired and untrimmed nucleotides, the amino acid sequences that produced functional peptides were filtered from the pool by searching for the vector-encoded sequence on each peptide end (starting with SNASF … or ending with … GAP). The resultant peptides bearing the vector sequences were grouped to form a new sequence list, which was used to create a Basic Local Alignment Search Tool (BLAST) database. The amino acid sequences of the 12 AHSV-4 proteins were used as reference and aligned against the new peptide sequence list using BLAST. Each peptide that matched was illustrated on a graph by adding the number of times an amino acid aligned at a position on the respective proteins. For this, the alignment table in CLC which shows where each peptide matches on the reference protein was used. A ‘per amino acid’ alignment graph was drawn in Microsoft^®^ Excel. The position and number of peptide matches were illustrated as peaks and compared to the DNA mapping.

## Results

### Enzyme-linked immunosorbent assay of horse antisera

To confirm the presence of AHSV-specific antibodies in the sera of the vaccinated horses, they were tested against purified AHSV-4 in ELISA. Sera from day 28 after administration of the vaccine gave the highest signals that had all decreased by between 23% and 70% by 52 (Figure 1). The day 0 signals indicated the sero-negative state of the horses prior to vaccination. IgG was purified from all the samples and used to select binders from the phage library (below).

**FIGURE 1:**
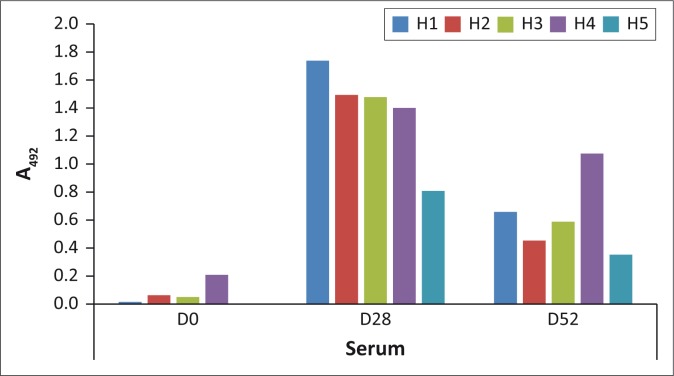
Enzyme-linked immunosorbent assay showing the antisera of the five horses (H1–5) collected pre-vaccination (day 0) and post-vaccination (day 28 and 52) reacting with purified African horse sickness virus-4. Each plotted value is an average of duplicate wells. Milk powder–negative control background signals were subtracted from the experimental data.

### Fragmented-genome library construction

**FIGURE 2:**
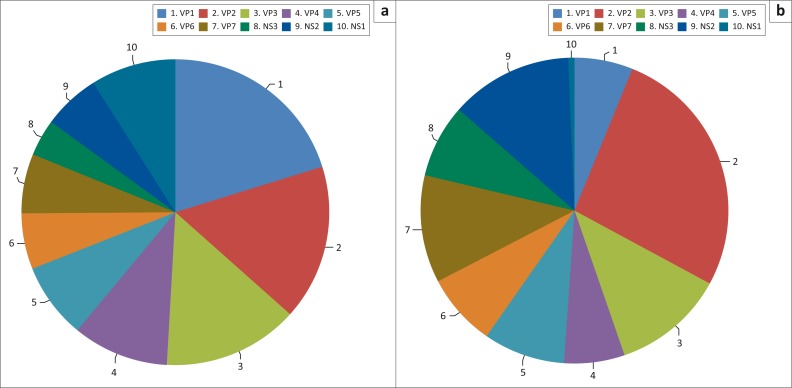
Proportional representation of the African horse sickness virus-4 genome segments according to (a) size and (b) actual number of clones in the primary unpanned fragmented-genome phage display library. Segments are colour coded and named according to their encoded protein.

To map the epitopes on AHSV-4 which were recognised by immune horse sera, a phage-displayed peptide library was constructed. It comprised 5.63 × 10^5^ clones with insert sizes ranging from 50 bp to 600 bp. This exceeded the minimum required size of 1.7 × 10^4^ clones to cover the ±19-kb AHSV genome with a 99% probability (Clarke & Carbon 1976). This took into account the fact that a fragment can be cloned in one of two orientations, with three reading frames on either the N- or C-terminus. This means that only 1/18 clones encoded a functional native AHSV-4 peptide (Jacobsson et al. 2003). To confirm coverage, an aliquot of the library was subjected to high-throughput sequencing and mapped to the entire AHSV-4 genome. Even though the depth of coverage varied across regions, there were no gaps. These results validated the theoretical calculations (Figure 1-A1a). Nevertheless, comparing the expected representation as a proportion of segment size and the actual numbers found by mapped sequences showed that not all the segments were represented equally in the library (Figure 2). Although the complete segment was represented, NS1 was the most underrepresented (Figure 1-A1a).

### Affinity selection of binding peptides

**FIGURE 3:**
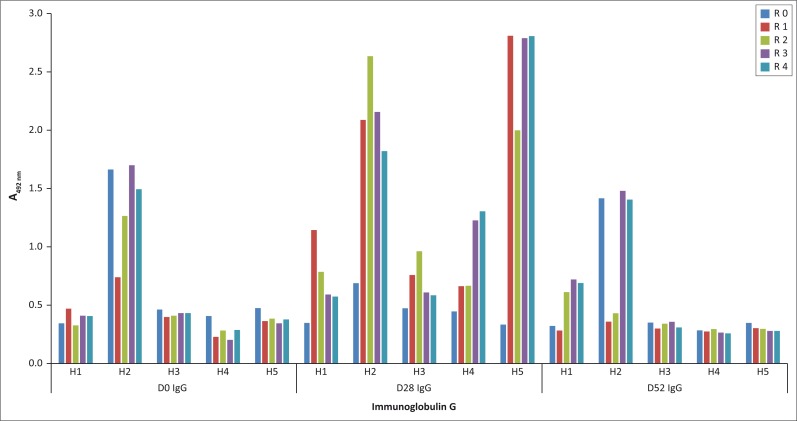
Enzyme-linked immunosorbent assay showing the pool of fusion phages in the unpanned library (R0) and phages selected during the four rounds of panning (R1 – R4) with day 0, 28 and 52 IgG of all five horses, reacting with their respective IgG. Each plotted value is an average of duplicate wells. Milk powder–negative control data have been subtracted from the experimental data.

The fragmented-genome library was individually affinity selected for binders using antibodies from each horse and at each indicated time point. Specific binding of the selected pools of phages to the respective IgGs was demonstrated with ELISA to show that the enrichment of phage pools was actually linked to their recognition by antibodies in the sera. There was an enrichment of phages with day 28 IgG especially for Horses 2 and 5 (Figure 3) but no enrichment with immunoglobulins from day 0 sera and very little with day 52 IgG. Only Horse 1 day 52 IgG-selected phages showed similar enrichment to those selected with Horse 1 day 28 IgG. Phage selected with Horse 2 IgG of both day 0 and day 52 time points showed ELISA signals of at least twice that of the other horses. However, the unpanned library resulted in similar signals, which may suggest the presence of TUPs, non-specific binding or anti-phage antibodies in Horse 2 serum.

### Characterisation of selected phages

It has been shown that comparing sequences from a selection round where there is no further enrichment with sequences from the unpanned library should give a clear change in binding pattern (Christiansen et al. 2015). Thus, pools of phages from panning round three for each time point were selected for further in silico characterisation. The DNA inserts were amplified by PCR, gel purified and sequenced. The data were received as 2 × 75-bp paired-end reads for each DNA fragment. The sequences were mapped to the ORFs of each of the AHSV-4 genome segments. Approximately 70% of the sequences mapped to the genome. The other 30% was either low quality or from untargeted sources and not taken into account.

**FIGURE 4:**
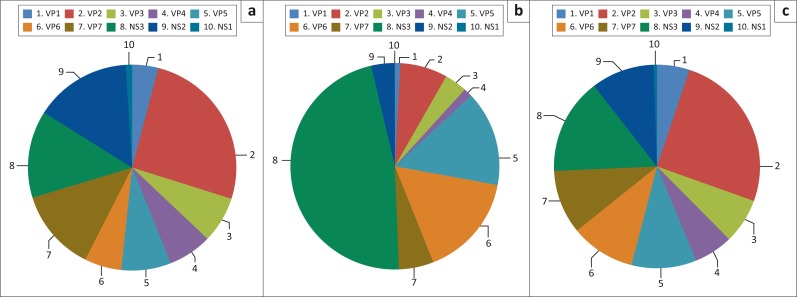
Proportional representation of paired-end sequence reads mapping to each segment after the third round of panning with anti-African horse sickness virus-4 IgG. (a) Day 0, (b) day 28 and (c) day 52. Data represent the average of the five horses for each time point. Segments are colour coded and named according to their encoded protein.

Theoretically, affinity selection of a display library using a population of antibodies creates a sequence distribution pattern biased towards the selected genome regions. When the sequences were mapped to the ORFs of AHSV-4 and the data for each time point pooled, a change in sequence distribution was observed (Figure 4). The figure only reflects the proportional sequence match per gene and not their position on the gene. The sequence distribution was in agreement with the phage-IgG ELISA signals since day 28 selected sequences showed the most notable change in proportional hits to each gene when compared to day 0 and day 52 selected sequences. Sequences mapping to the ORF encoding for NS3, VP5 and VP6/NS4 increased by the largest amount on day 28 and had decreased to nearly day 0 levels by day 52.

**FIGURE 5:**
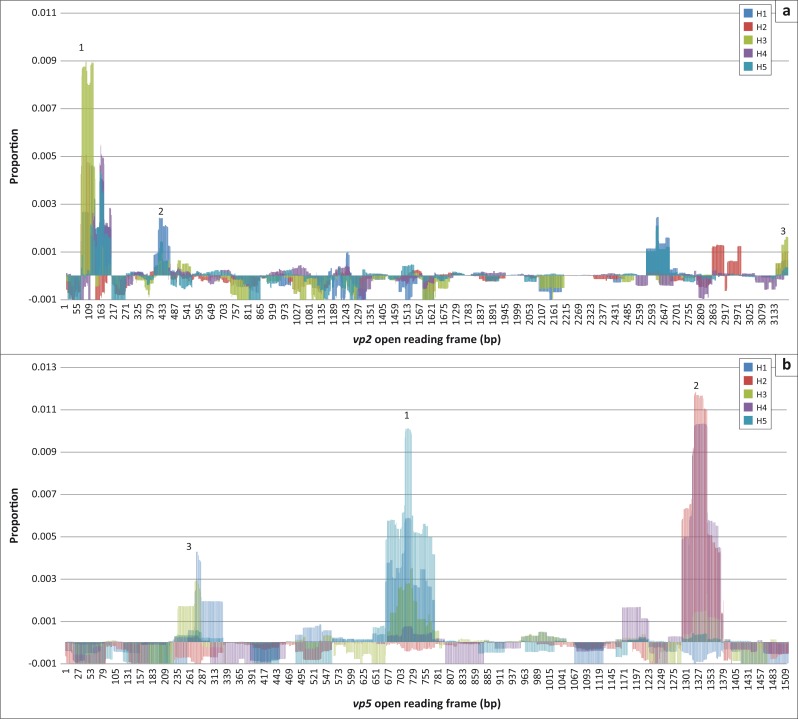
Day 28 IgG-selected VP2 (a) and VP5 (b) sequences, of all five horses, converted to matches per nucleotide position as a proportion of the total nucleotide matches per gene segment. Numbers represent the identified potentially antigenic regions. Data were normalised by subtraction of day 0 matches.

The sequences identified with day 28 IgG were further analysed to determine the regions on the individual genome segments with the highest number of overlapping sequences and thus potential antigenicity. In contrast to the complete coverage of a genomic segment prior to panning, sequences mapped only to specific positions on each segment (Figure 1-A1b). The number of hits at a specific position was illustrated as a proportion of total hits for that genomic segment. For easy comparison, the results were combined on a single graph for all horses. For example, VP2 data of each horse were plotted on one chart (Figure 5a) and the same for VP5 (Figure 5b). The regions that were recognised by the antibodies from all or most of the horses were focussed on in this study. Very few sequences mapped to the segment encoding NS1, which was therefore excluded from subsequent characterisation.

**FIGURE 6:**
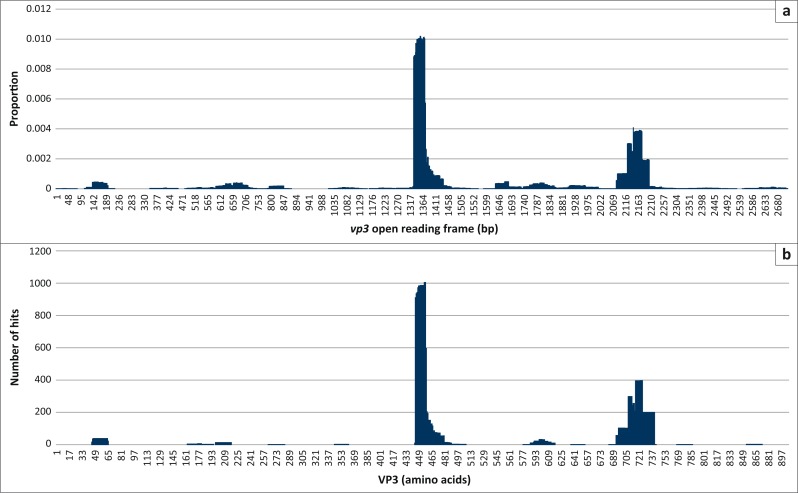
A comparison of the pattern of nucleic sequences mapping to the gene and the deduced amino acid alignments of the selected peptides using the day 28 VP3 data of Horse 2 as an example. (a) Nucleic acid mapping and (b) amino acid mapping.

**FIGURE 7:**
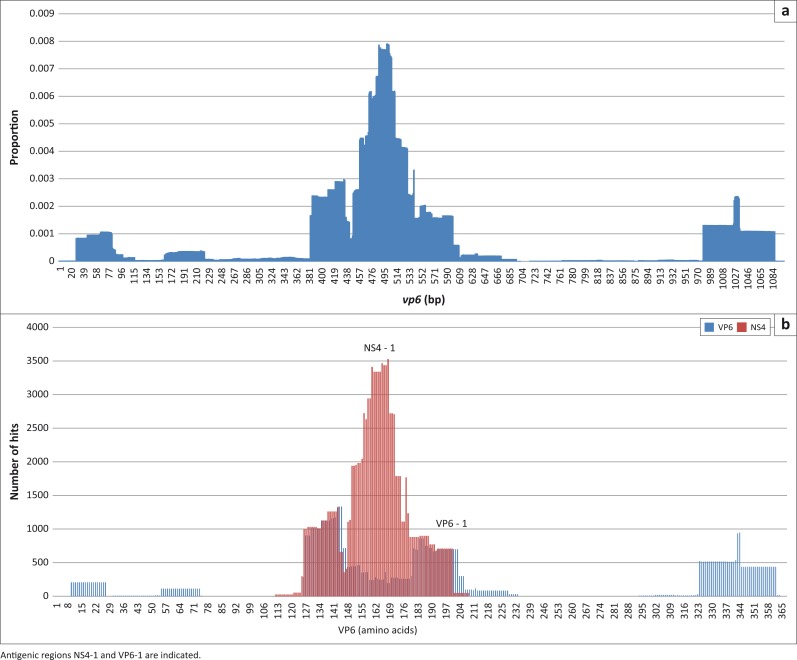
A comparison of the pattern of nucleic acid sequences mapping to the gene and the deduced amino acid alignments of day 28 VP6 data of Horse 2. The translated sequences are matched to either the VP6 or the NS4 amino acid sequence. NS4 is in the +2 reading frame from bp 197 to 631 but positioned with VP6 for ease of comparison. (a) Nucleic acid mapping and (b) amino acid mapping.

Usually with phage-displayed peptides, the inserts of single clones are sequenced, translated and then mapped to the amino acid sequence of the target protein. The in silico analysis described here was done with nucleic acid sequences. For phage-displayed peptides derived from randomly fragmented DNA, there is a possibility that the DNA insert is expressed as a functional fusion-phage protein but as a non-native peptide. To determine whether the nucleic acid matches described above were from phages expressing native AHSV-4 peptides, the sequences were also translated and aligned to the amino acid sequences of each protein. The translated sequences contained the primer/vector sequences and were selected based on the vector-encoded pVIII amino acids at the cloning site. Sequencing errors such as insertions and deletions could affect the correct translation of reads and result in discarding of reads/matches when the amino acid sequences are filtered for correct reading frame and thus the matching pattern. The translated sequences were aligned to the amino acid sequence of the AHSV proteins in a similar pattern as the nucleotide sequence mapping, for example, VP3 of Horse 2 (Figure 6). Because the patterns were similar, the majority of sequences after panning were expressed on the phage as native AHSV peptides. This validates the less cumbersome route of using nucleotide sequences to map to the targets. However, there are exceptions; the gene segment coding for VP6 contains a second open reading frame in both BTV and AHSV (Belhouchet et al. 2011; Zwart et al. 2015). This is a small, non-structural protein, named NS4. In this case, it is essential to look at the amino acid mapping to distinguish VP6 and NS4 matches (Figure 7). For the AHSV-4 used in this study, NS4 is coded in the +2 reading frame from nucleic acid position 197 bp to 631 bp. The DNA region coding for both proteins contains a major antigenic region recognised by the antibodies from all five horses, which is in fact an NS4 epitope (NS4-1). The regions flanking NS4-1 are VP6 epitopes but only VP6-1 is common between all the horses and not recognised by day 0 antibodies. In addition, segment 10 that encodes NS3 also contains a second reading frame (S10-ORF2; Stewart et al. 2015). In this case, less than 0.1% of the hits are in the S10-ORF2. Thus, the NS3 antigenic region remains the most recognised by the antibodies.

Seventeen antigenic regions recognised by day 28 IgG from most horses were identified (Table 1). They were between 18 and 60 amino acids (aa) in length and mostly on the outer capsid proteins VP2 and VP5 (three each). Two antigenic regions were identified on each of the inner core VP7, the sub-core VP3 and non-structural protein NS3. One region was identified by IgG from all horses on each of the minor structural proteins VP1, VP4 and VP6 and the non-structural proteins NS2 and NS4. Most of these regions were also identified by day 52 IgG with the exception of VP2-2.

**TABLE 1:** Potential antigenic regions on African horse sickness virus-4 proteins.

Peptide	Amino acid position	Size (aa)	Recognised by day 52 IgG
VP1-1	1140–1166	26	Yes
VP2-1	20–63	33	Yes
VP2-2	123–150	27	No
VP2-3	1036–1054	18	Yes
VP3-1	435–468	33	Yes
VP3-2	693–730	37	Yes
VP4-1	123–160	37	Yes
VP5-1	200–260	60	Yes
VP5-2	430–460	30	No
VP5-3	80–110	30	Yes
VP6-1	181–204	23	Yes
VP7-1	207–235	28	Yes
VP7-2	1–20	19	Yes
NS2-1	190–213	23	Yes
NS3-1	1–50	49	Yes
NS3-2	56–83	27	Yes
NS4-1	84–114	30	Yes

aa, amino acids.

## Discussion

Identifying antigenic regions on viruses and other pathogens remains important in vaccine and immunodiagnostic development. In this study, B-cell antigenic regions of AHSV-4 recognised by polyclonal sera from immunised horses were identified using phage display in conjunction with high-throughput sequencing of the selected sequences, in an approach similar to the PROFILER method (Domina et al. 2014). This polyclonal pool of antibodies induced by the vaccination represents the complete antibody response that may contain neutralising antibodies.

Analyses were focussed on the phage-displayed peptides selected using antibodies from day 28 when the immunoglobulin levels were at their peak. The panning with day 52 IgG yielded less enrichment of binders than day 28 IgG (Figure 3) even though there were anti–AHSV-4 antibodies in the day 52 serum (Figure 1).

The overlapping nucleotide sequences mapped predominantly to segments encoding NS3, VP6/NS4 and VP5. Anti-AHSV NS3 antibodies have been found to occur at low concentrations in infected horses using immunoblotting (Laviada et al. 1993), while high concentrations of anti-VP6 and VP5 antibodies were similarly detected using horse serum from early-stage infection (Martinez-Torrecuadrada et al. 1997). Not all studies on AHSV antigenicity concur. The apparent differences may, among other reasons, be because of the time the serum was collected and methods used to determine antigenicity. A possible factor may be that the long fragments displayed in this study may adopt secondary structures and thus mimic discontinuous epitopes (Fehrsen et al. 2005). This of course would not be the case with a linear or denatured peptide. It was not determined whether the epitopes on NS3, VP6/NS4 and VP5 induce neutralising antibodies. However, in a related orbivirus, candidate vaccines without NS3 or with non-functional VP6 protected sheep after challenge with a virulent BTV strain (Feenstra et al. 2014; Matsuo, Celma & Roy 2010; Matsuo & Roy 2009; Matsuo et al. 2011). This may suggest that NS3 and VP6 of AHSV do not induce neutralising antibodies. To account for the immuno-dominance of NS3 and VP6/NS4, it is possible that the virus uses these proteins to deceive the immune system during infection, as described for HIV1 using gp120, termed ‘deceptive imprinting’ (Nara & Garrity 1998). The virus uses the proteins to divert the immune system’s attention from the immunopathogenic hot spots on proteins or replication machinery, thus evading neutralisation. On the other hand, AHSV VP5 expressed in insect cells induced antibodies that neutralised the virus in a plaque reduction assay (Martinez-Torrecuadrada et al. 1999) and in combination with VP2 and VP7 protected horses against AHS (Martinez-Torrecuadrada et al. 1996). Thus, the VP5 epitopes identified here might be able to induce neutralising antibodies.

Although multiple regions were selected with the different horse IgGs, only those that are selected by IgG from all horses are likely to be of interest. These regions are not unique to a particular animal and thus may be useful in developing a universal recombinant vaccine or diagnostic reagent. The regions with the most peptide sequences selected by phage display were NS3-1 (49 aa), NS3-2 (27 aa) and NS4-1 (30 aa). NS3-1 contains a proline-rich area (Huismans et al. 2004). A proline-rich region was essential for plasma membrane targeting and viral release in the Ebola and Marburg virus matrix protein VP40 (Reynard et al. 2011). NS3-1 could be involved in membrane binding and directing viral egress, thus being exposed to the immune system during virus replication. The other dominant region identified is contained on the VP6 encoding segment. This area maps to the NS4 amino acid sequences. Because these NS3 and VP6 (and in effect NS4) proteins have previously been found to be non-neutralising and not critical for virus propagation, removing them from a vaccine construct may increase vaccine efficacy (Stalhammar-Carlemalm et al. 2007).

Three potentially antigenic regions were mapped on VP5. Both VP5-1 and VP5-3 form part of regions found to react with serum from an AHSV-4–infected horse in immunoblots. VP5-3 contains a neutralising epitope (position 85–92) as recognised by a mouse monoclonal antibody (Martinez-Torrecuadrada et al. 1999). VP5-2 was not recognised by horse antibodies in an immunoblot; thus, it is possible that this region, identified by phage display, represents part of a discontinuous epitope.

Three antigenic regions were identified on VP2 (33-, 27- and 18-aa long), with the first two near the N-terminal half and one at the C-terminus. These are not the same as previously identified epitopes mapped by others using horse antibodies by both phage display and PEPSCAN. It is possible that the longer phage-displayed peptides in this study (up to 200 aa compared to 100 aa) allow formation of secondary structures, which can reveal additional antigenic regions. The region between amino acid positions 200 and 432, identified by only two horses in this study, was described as being dominant using mouse monoclonal antibodies, rabbit and horse polyclonal antibodies and PEPSCAN (Bentley et al. 2000; Martinez-Torrecuadrada et al. 2001). For VP2, the advantage of the large amount of data yielded by high-throughput sequencing of the released pool of phages instead of the traditional clone picking is clearly evident.

Two potential antigenic regions were mapped on VP7, one towards the centre (28 aa) and the other at the N-terminus (19 aa). These regions may have some relevance to neutralisation because the addition of VP7 in a mixture with VP2 and VP5 increased immunological protection of horses (Martinez-Torrecuadrada et al. 1996). Furthermore, AHSV VP7 purified from BHK-infected cells was able to protect BALB/c mice against lethal challenge, although it induced low antibody titres as detected with ELISA, suggesting that such protection could conceivably also involve cell-mediated responses (Wade-Evans et al. 1997). This suggestion was supported recently where VP7 induced equine cytotoxic T-cell responses in vivo (Faber et al. 2016). One region was mapped at the centre of NS2 (27 aa). In another study, recombinant NS2 and VP7 of AHSV serotype 3 reacted with hyperimmune guinea-pig sera to all nine serotypes and horse antisera to seven attenuated AHSV serotypes (Bremer et al. 1994) which indicates both NS2 and VP7 contain conserved epitopes. The antigenicity of VP1, 3 and 4 remain largely unexplored. Peptides representing these proteins were selected at a low frequency in this study.

The size of an epitope varies between 5 and 15 binding residues (Kringelum et al. 2013). The regions identified in this study are larger, each of which could possibly contain shorter minimal binding areas or are able to adopt a secondary structure and represent discontinuous epitopes as in the protein’s native form.

Phage display in conjunction with high-throughput sequencing methods have shown to be valuable tools to get a comprehensive antigenic profile of proteins. It was possible to confirm some of the antigenic regions identified by other methods, for example, by PEPSCAN. As an alternative and to avoid potential bias from the display system used in this study, a bacteriophage T7 system can be used where the phages are released by lysis and not secreted through the cell membrane. This system was used to clone the complete human virome, made synthetically and codon optimised for *E. coli* expression. Immuno-precipitation was used for selection thus avoiding solid-phase selection used in this study. Although it is more expensive to set up than the fragmented-genome approach, together with high-throughput sequencing this method can also yield a wealth of data (Xu et al. 2015).

## Conclusion

In conclusion, 17 horse-specific potential antigenic regions on both structural and non-structural proteins of AHSV-4 were identified using phage display combined with high-throughput sequencing. The antibodies used to identify the regions were from horses vaccinated with the serotype 4, which is included in the commercial AHSV vaccine, which is known to induce protection against African horse sickness. It needs to be confirmed whether these regions are in themselves involved in immunological protection. Once confirmed as being potentially protective B-cell epitopes, they could conceivably be combined with suitable T-cell epitopes (Faber et al. 2016) to form a vaccine construct capable of evoking both humoral and cellular immunity. Apart from vaccine development, B-cell epitopes on NS2, VP6, NS3 and VP7 may also assist in the development of diagnostic tests.

In addition to identifying antigenic regions elicited by a vaccine administered to the actual host animal, this study has assisted in developing approaches that can facilitate analysis of the large volume of phage display data. This approach is likely to be useful in monitoring the global antibody response in animals during vaccine trials.

## Figures and Tables

**FIGURE 1-A1:**
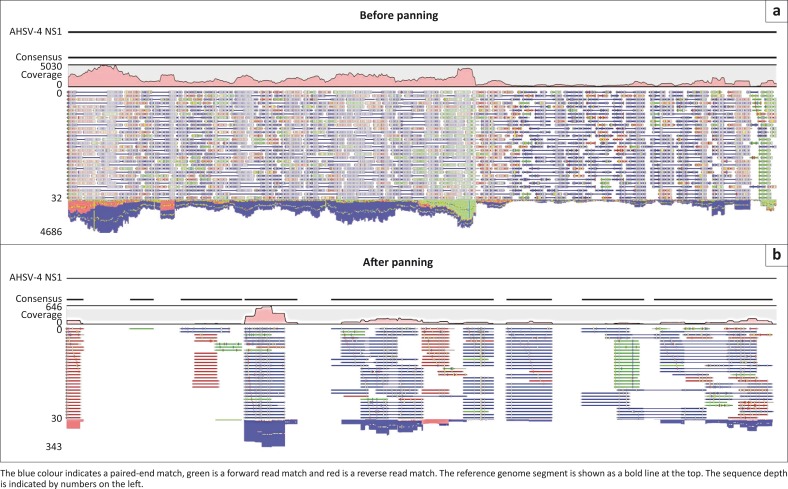
An example of a CLC Genomics Workbench read mapping representation of sequenced inserts mapping to the African horse sickness virus-4 open reading frames segment encoding NS1. The sequences from the unpanned library (a) cover the entire gene without any gaps. The sequences from round three phages selected with day 28 IgG (b) has gaps, indicating that only regions of interest have been selected.
